# FAIR SHARING OF THE BLSA DATA ECOSYSTEM

**DOI:** 10.1093/geroni/igac059.1231

**Published:** 2022-12-20

**Authors:** Michael Griswold, James Henegan, Chad Blackshear, Ann Moore, Thomas Smith, Deric McGowan, Eleanor Simonsick, Luigi Ferrucci

**Affiliations:** The MIND Center at UMMC, Jackson, Mississippi, United States; UMMC MIND Center, Jackson, Mississippi, United States; UMMC MIND Center, Jackson, Mississippi, United States; TGB NIA NIH, Baltimore, Maryland, United States; NIA, Baltimore, Maryland, United States; NIA, Baltimore, Maryland, United States; National Institute on Aging, Baltimore, Maryland, United States; National Institute on Aging, Baltimore, Maryland, United States

## Abstract

The BLSA is an invaluable resource for the study of human aging with uniquely rich measures taken longitudinally on a continuous replenishment cohort since 1958. Tremendous interest has been expressed by the United States and international research communities in expanded access to and use of BLSA data to address emerging scientific questions. Here, we will describe recent study leadership initiatives into: (1) making the BLSA data more FAIR (Findable, Accessible, Interoperable, and Reusable), (2) growing a BLSA data ecosystem that enables scalable sharing, and (3) leveraging and developing platforms for accessing the ecosystem. Lastly, we will demonstrate current platform functions that empower researchers to find and access BLSA data, metadata, protocols, code and supporting materials to make new discoveries.

